# Supplementary material for: Stacking Model‐Based Classifiers for Dealing With Multiple Sets of Noisy Labels
Source: Biom J. 2025 Mar 12;67(2):e70042. doi: 10.1002/bimj.70042 (PMC11898607; doi:10.1002/bimj.70042)
Supplement: Supplementary file 1 — Supporting Information [file BIMJ-67-e70042-s001.zip › density-based-ensemble-model-main/competitors_real_data/netlab-master/netlab-master/help/gtmem.htm]

Netlab Reference Manual gtmem


# gtmem

## Purpose

EM algorithm for Generative Topographic Mapping.

## Synopsis

```
[net, options, errlog] = gtmem(net, t, options)
```

## Description

`[net, options, errlog] = gtmem(net, t, options)` uses the Expectation
Maximization algorithm to estimate the parameters of
a GTM defined by a data structure `net`.
The matrix `t` represents the data whose expectation
is maximized, with each row corresponding to a vector. It is assumed
that the latent data `net.X` has been set following a call to
`gtminit`, for example.
The optional parameters have the following interpretations.

`options(1)` is set to 1 to display error values; also logs error
values in the return argument `errlog`.
If `options(1)` is set to 0,
then only warning messages are displayed. If `options(1)` is -1,
then nothing is displayed.

`options(3)` is a measure of the absolute precision required of the error
function at the solution. If the change in log likelihood between two steps of
the EM algorithm is less than this value, then the function terminates.

`options(14)` is the maximum number of iterations; default 100.

The optional return value `options` contains the final error value
(i.e. data log likelihood) in
`options(8)`.

## Examples

The following code fragment sets up a GTM, initialises
the latent data sample and RBF
parameters from the data, sets the options and trains the model.

```
% Create and initialise GTM model
net = gtm(latentdim, nlatent, datadim, numrbfcentres, ...
   'gaussian', 0.1);

options = foptions;
options(1) = -1;
options(7) = 1;    % Set width factor of RBF
net = gtminit(net, options, data, 'regular', latentshape, [4 4]);

options = foptions;
options(14) = 30;
options(1) = 1;
[net, options] = gtmem(net, data, options);
```

## See Also

`gtm`, `gtminit`

---

**Pages:**
Index


---

Copyright (c) Ian T Nabney (1996-9)
